# Opportunities to Improve the Implementation of Relational Health Interventions for Children and Families: A Secondary Analysis of a Scoping Review

**DOI:** 10.1007/s10567-026-00567-5

**Published:** 2026-04-30

**Authors:** Natasha Pearce, Donna Cross, Jacinta Francis, Jonathan H. Sae-Koew, Alexander M. Godley, Caitlin Attwell, Tracy Evans-Whipp, Jacqueline Allen, Ross Homel, Craig A. Olsson, the Australian Early Relational Health Network, Jacqueline Allen, Cath Chamberlain, Juli Coffin, Donna Cross, Tracy Evans-Whipp, Alex Fischer, Jacinta Francis, Matthew Fuller-Tyszkiewicz, Rebecca Glauert, Melissa Green, Ross Homel, Primrose Letcher, Jacqui A. Macdonald, Kayla Mansour, Jennifer McIntosh, Shaun Mclaws, Siobhan M. O’Dean, Craig Olsson, Felicity Painter, Natasha Pearce, Naomi Priest, Lisa Ritland, Tim Slade, Liz Spry, Sarah Whittle, Lu Zhang, Stephen R. Zubrick

**Affiliations:** 1 The Kids Research Institute Australia, Perth Children’s Hospital, Nedlands, WA Australia; 2 The University of Westen Australia, Perth, WA Australia; 3 Edith Cowan University, Joondalup, WA Australia; 4 Faculty of Health, SEED Centre for Lifespan Research, School of Psychology, Deakin University, Burwood, VIC Australia; 5 Griffith Criminology Institute, Griffith University, Nathan, QLD Australia; 6 Centre for Adolescent Health, Department of Paediatrics, Murdoch Children’s Research Institute, The University of Melbourne, Royal Children’s Hospital Campus, Parkville, VIC Australia

**Keywords:** Relational health, Childhood and adolescence, RCT-evaluated, Universal interventions, Implementation outcomes, Fidelity

## Abstract

Despite the availability of universally delivered evidence-based relational health interventions, their wide-scale translation into practice is slow and unsustained. Measuring implementation outcomes within intervention trials is critical to understand their effectiveness and inform future scaling. This study aimed to determine the extent to which universally delivered relational health interventions measured and reported on implementation outcomes to inform their effectiveness. A secondary analysis of a scoping review examined the extent to which 123 RCT-evaluated relational health intervention studies referred to (1) implementation related theories or frameworks; (2) implementation data collection methods; (3) implementation measures, and; (4) each of twelve implementation outcomes (acceptability, adoption, appropriateness, cost, feasibility, fidelity-adherence, fidelity-dose, fidelity-delivery, fidelity-responsiveness, fidelity-differentiation, penetration and sustainability). The type of implementation trial was also assessed. Seventeen percent of studies reported implementation outcomes as part of the intervention main effects, with the remaining studies (83%) reporting implementation outcomes as a minor sub-analysis (52%), in their discussion only (21%) or not at all (10%). The most frequently reported outcomes were fidelity measures of dose and adherence. Almost a half of the twelve implementation outcomes were not addressed. No studies described an implementation-related theory or framework, and all used non-standardised measures. Greater attention to measuring and reporting implementation outcomes within relational health intervention trials is needed to ensure that critical evidence for replicability and scalability is available to those who are considering implementation in practice. This would facilitate improvements in both intervention implementation and sustainability and importantly, relational health outcomes for children and families.

**Supplementary Information:**

The online version contains supplementary material available at 10.1007/s10567-026-00567-5.

## Introduction

The most proximal process of human development involves interaction between caring adults and the children and young people within their care. Within these interactions, key knowledge, skills, and abilities transmit from one generation to the next (Bronfenbrenner, 2001, 2005). Promoting quality interactions in every microsystem of early development – family, school, peer, digital, community and more – is paramount to growth and development. The term ‘relational health’ is used to define the types of interactions between care providers (typically adults) and those they care for (children and young people) that enable the realisation of human psychological, social, cognitive and human potential (Black et al., 2017). Mutuality of trust between carers, children and young people is considered the most fundamental currency of relational health and the secure base from which development of human capabilities is best realised across the life course (Ainsworth, 1979; Erikson, 1964; Stern, 1985).

To the contrary, relational adversity, such as family neglect or abuse, low quality education and care or living in unsafe neighbourhoods, places children and young people at significantly heightened risk of developing social, emotional, behavioural and mental health problems (Clifford et al., 2023; Herd & Kim-Spoon, 2021; Shonkoff et al., 2012). Such early relational experience can influence developmental trajectories well into adulthood, including transitions to parenthood (Painter et al., 2025; Robson et al., 2020; Ttofi et al., 2012), with even longer-term impacts on next generation offspring (Macdonald et al., 2025). Interventions that optimise early relational health experiences may help to break the cycles of disadvantage for children and families (Paul Ramsay Foundation, 2022).

There are a range of universally delivered evidence-based interventions that target risk and protective factors important to relational health outcomes at different levels of the social ecology around children and young people (Allen et al., 2026; Fuller-Tyszkiewicz et al., 2026; O’Dean et al., 2025). These interventions have been designed to support healthy relationships by promoting quality peer relationships, strengthening social and emotional competence and wellbeing, and intervening on parenting styles to build secure parent–child attachments (Branco et al., 2022; Durlak et al., 2011; Gaffney et al., 2019; Taylor et al., 2017b). Whilst targeted interventions to support individuals with identified high need are critical, universal interventions (delivered to everyone within a defined group) can offer increased population reach, as well as prevention and early intervention opportunities that reduce health care burden and increase intervention cost efficiencies (Abou Jaoude et al., 2024; Greenberg & Abenavoli, 2016). For example, universal school-based social and emotional learning (SEL) programs such as PATHS (Shi et al., 2022) and Friendly Schools (Cross et al., 2019) strengthen empathy, cooperation and conflict-resolution skills, with meta-analyses showing broad improvements in social functioning and reduced peer conflict such as bullying (Durlak et al., 2011). Long standing universal parenting programs such as Triple P (Sanders, 2023) and Incredible Years (Gardner & Leijten, 2017) have shown effectiveness at improving a broad range of child, parent and family outcomes.

However, despite this abundance of effective universal interventions, many fail to achieve their potential impact due to poor implementation (Britto et al., 2018). Further, the wide-scale translation of relational health interventions into clinical and community settings is typically slow and unsustained, with only a small portion reaching children and families with sufficient ‘dose’ to realise benefits (Cook & Farley, 2019). This research-to-practice gap is likely due to a poor understanding of the implementation barriers and processes required to translate interventions effectively into real life policy and practice (Durlak & DuPre, 2008; Kessler & Glasgow, 2011; Michie et al., 2009).

Robust empirical evidence suggests the quality of intervention implementation is critical to improving health and wellbeing outcomes, including relational health outcomes. Durlak and DuPre (2008) identified over 500 quantitative child and adolescent studies that link the level of implementation to prevention intervention outcomes. Barnett (1995) found in a review of 36 public programs that the impacts of empirically-based early childhood interventions were affected by the quality of implementation. Ttofi and Farrington (2011) found that program duration and intensity (dose) for students and teachers was one of the main factors associated with a significant decrease in rates of bullying and victimisation. In their meta-analyses of studies assessing the effectiveness of the PATHS curriculum, Shi et al. (2022) found that dosage was the most predominant factor in determining the effects of PATHS. Other researchers have also found an association between implementation quality and intervention outcomes such as improved social and emotional competencies and academic performance (Askell-Williams et al., 2013; Dix et al., 2012). Given that the effectiveness of interventions strongly depends on the quality of their implementation, the reporting of intervention main effects should always include a description and measurement of its implementation (Baumann et al., 2016). Evaluating implementation outcomes and processes alongside relational health outcomes is critical to understanding why, and under what conditions, interventions are effective and to eliminate Type 3 error (i.e., rejecting an intervention as unsuccessful when its failure may be due to poor implementation) (Kaur & Stoltzfus, 2017).

Effectiveness trials often result in lower effect sizes than decontextualised highly controlled efficacy trials due to the intention to test the intervention under real-world and therefore, uncontrolled conditions, and address potential implementation challenges (Glasgow et al., 2003). For example, interventions under real-world conditions need to have the flexibility to ‘fit’ the intended family, community or service system context and need to be delivered by skilled practitioners rather than researchers (May et al., 2016). Implementation quality and intervention effects can also become increasingly diluted the more the intervention is scaled out to other settings or scaled up to larger populations (Britto et al., 2018). Research has begun to answer the questions of ‘why’ and ‘what’ to do to address children’s and adolescents’ relational health but ‘how’ to apply it effectively in different contexts and sustain it in practice, is still largely unknown (Aboud et al., 2018; Dumitriu et al., 2023). Hence, it is critical to assess and understand intervention implementation processes and outcomes during testing within RCTs, particularly within effectiveness trials, to inform future scaling and increase the speed of translation into routine practice.

Implementation science is a rapidly growing field of research that determines the key contributors to the effective implementation of evidence-based interventions and how implementation processes can be effectively measured (Chambers & Emmons, 2024). It has amalgamated the use of existing theories and frameworks to plan and evaluate implementation outcomes and processes, for example, The Diffusion of Innovations Theory (Rogers & Rogers, 2003), Normalisation Process Theory (Murray et al., 2010), the PRECEED-PROCEED model (Lawrence et al., 2022), and RE-AIM (Glasgow et al., 2019). The Consolidated Framework for Implementation Research (CFIR) is a determinants framework developed by combining constructs from a range of theories to map both the characteristics of the intervention itself and contextual factors (barriers and facilitators) at varying levels of implementation, that may influence implementation success (Damschroder et al., 2022). Greater use of theories and frameworks is needed to strengthen RCT study designs and support the measurement and interpretation of both implementation and intervention effects, to increase the pace of translation to practice and to progress the field of implementation science (Moullin et al., 2020; Nilsen, 2015). 

Evaluating the implementation of interventions requires clearly defined and measurable implementation outcomes and assessment of the process and quality of intervention delivery. Implementation outcomes are distinct from intervention outcomes to change health and wellbeing; they are the effects of purposeful implementation strategies, such as capacity building, employed to deliver and integrate the intervention into new settings. Proctor and colleagues (2009, 2011) define eight measurable implementation outcomes now widely used in intervention research: (1) Acceptability: the perception among stakeholders that a specific intervention or practice is agreeable or satisfactory; (2) Adoption: the initial decision or ‘uptake’ to undertake an evidence-based intervention or practice; (3) Appropriateness: the perceived fit, relevance or compatibility of the intervention or practice for a given setting; (4) Feasibility: the extent to which a new intervention or practice can be successfully used or conducted within a given service or setting; (5) Cost: the cost of the intervention, the implementation strategies and any additional costs required to support its implementation; (6) Fidelity and Adaption: the degree to which an intervention or practice was implemented as it was prescribed in the original protocol or adapted by the providers; (7) Penetration: the ‘reach’ or integration of an intervention or practice within a setting and its subsystems; and (8) Sustainability: the extent to which a newly implemented intervention or practice is maintained or institutionalised within a setting’s ongoing operations.

Each of these eight outcomes can function as indicators of implementation success, proximal indicators of implementation processes, and as key intermediate outcomes, distinct from policy and practice or participant health and social outcomes (Proctor et al., 2011). These outcomes can also be applied to aspects of both the intervention (e.g. content) and its delivery (e.g. curriculum lessons) and also to specific implementation strategies, such as training and coaching, or system targeted strategies, such as establishment of a community coalition or service workforce positions. In particular, fidelity outcome measures help researchers to identify and monitor real-life processes and complexities and ensure the effects can be attributed to the intervention as described (Greenberg, 2004; Lendrum & Humphrey, 2012). Without assessing the fidelity of the intervention and its implementation, it is difficult to determine the balance between useful context adaptation and drift from effective practice. Five dimensions of fidelity are commonly assessed: adherence (intervention implementation as designed); dosage (amount of intervention delivered to or received by participants); quality of intervention delivery (effectiveness of intervention strategies delivery); participant responsiveness (level of engagement in and receptiveness to the intervention); and program differentiation (whether essential elements of the intervention implementation distinguish treatment from comparison groups when evaluating its efficacy) (Carroll et al., 2007; Gearing et al., 2011).

Psychometrically valid and reliable measures of implementation outcomes enable cross-study comparison and standardisation of constructs. Although instrumentation issues in implementation science are recognised and said to be in an early stage of development, (Lewis et al., 2015a; Martinez et al., 2014), validated measures are available (e.g., Acceptability of Intervention Measure (AIM), Intervention Appropriateness Measure (IAM), and Feasibility of Intervention Measure (FIM); (Weiner et al., 2017) and resources such as repositories (e.g., SIRC: Society of Implementation Research Collaboration) support researchers to access measures with quality ratings (Lewis et al., 2015b). These measures often need to be tailored to the specific intervention, however, their use and the reporting of any adaptations and additional validity and reliability testing is paramount to building standardised measurements for the field and reducing the gap between what intervention is intended and what is delivered (Mettert et al., 2020).

The use of hybrid effectiveness-implementation studies that assess fidelity and other implementation outcomes simultaneously with intervention effectiveness outcomes can improve our understanding of implementation processes and impact on their efficacy and effectiveness (Wolfenden et al., 2021). Hybrid studies can optimise intervention delivery and impact by ensuring sufficient intervention’dose’ is delivered to benefit children and families and monitoring fidelity and contextual adaptation to maintain its effectiveness while meeting local needs. These studies can also accelerate the speed of translation into practice and the scaling of interventions in diverse contexts by reducing the likelihood of incorrect conclusions about intervention success, particularly by distinguishing between the intervention’s implementation and its effectiveness and identifying why and how the intervention succeeded or failed. A range of research and evaluation study designs are being used to provide a deeper understanding of the translation processes required for interventions to be scaled and sustained in community and service systems (Brown et al., 2017).

Three types of hybrid studies are defined by Curran et al., (2012, 2022) as: Type 1: testing of intervention strategies on health and social outcomes while observing and gathering information on implementation; Type 2: dual testing of intervention and implementation strategies and outcomes; and Type 3: testing of implementation strategies on implementation related outcomes while observing and gathering information on the intervention’s impact on health and social outcomes. When established implementation frameworks, such as CFIR and RE-AIM, are used in hybrid studies to identify barriers and evaluate implementation outcomes, interventions can be properly assessed at the end of a trial to determine if they are suitable for wider dissemination and scaling, while also greatly enhancing sustainability and impact.

This paper is part of a review series on early relational health highlighting the need to build a relational health research and practice agenda that extends thinking across the life span and generations (Paul Ramsay Foundation, 2022). Specifically, we conducted a secondary analysis of reported implementation outcomes within published articles identified by Allen et al. (2026) of RCTs testing universal relational health interventions for children and young people (4–24 years). Our purpose was to understand to what extent RCTs of universally delivered relational health interventions assess implementation-related outcomes to explain the intervention’s effectiveness, and to inform future scaling and practice. We addressed this by describing (a) the inclusion of implementation outcomes in trial design (e.g., type of effectiveness-implementation study); b) the reporting of 12 core implementation outcomes, including five sub-dimensions of fidelity; (c) the implementation outcomes data collection methods and measures used; and (d) the use of theories and frameworks to guide intervention implementation.

## Methods

### Study Design and Data Sources

This study revisits the scoping review of RCTs of universally delivered interventions designed to promote relational health (interventions that support healthy relationships) from childhood to young adulthood (4–24 years) conducted by Allen et al. (2026). The authors defined relational health for the review as: “the growth-promoting qualities of all forms of mutually influential relations in a child’s or young person’s developmental system” (Allen et al., 2026). The RCTs identified in the scoping review evaluated interventions that were: (1) at any level of the human social ecology; (2) at any stage of the early life course to age 24; and (3) able to be applied universally to populations or proportionate to need, within universal service systems. RCTs were chosen as they provide the best standard of evidence for a causal effect of an intervention on relational health outcomes. Although this excludes other research designs that may have greater policy relevance (e.g. large quasi-experimental trials), limiting the review to RCTs kept the number of studies to a manageable level. The scoping review identified 123 peer reviewed papers published prior to August 2025; for full details of the methodology see Allen et al. (2026).

This secondary analysis explored the extent to which these RCTs considered and reported on intervention implementation, as broadly recommended by the SPIRIT 2013 statement (items 11a–d) (Chan et al., 2013, 2025) and more precisely, by the TIDieR checklist 2014 (items 2–12) (Hoffmann et al., 2014) and the Standards for Reporting Implementation Studies (StaRI) (Pinnock et al., 2017). The inclusion of implementation outcomes in trial design (Type 1, 2 or 3 effectiveness-implementation trial (Curran et al., 2022); the application of implementation theories and frameworks as identified in a recent systematic review (Birken et al., 2017); assessment of implementation outcomes using Proctor et al.’s (2011) eight pre-defined implementation outcomes (acceptability; adoption; appropriateness; feasibility; cost; fidelity/adaption; penetration; and sustainability); and five common measures of fidelity (adherence; dose; quality of delivery; participant responsiveness; and program differentiation) were investigated (Carroll et al., 2007; Gearing et al., 2011) (See Table 1 for definitions). Implementation outcome data collection methods and measures were also documented.

**Table 1:** Taxonomy of 12 implementation outcomes. Adapted from Carroll et al. (2007); Gearing et al. (2011); Pinto et al. (2024); Proctor et al. (2011)

Implementation Outcome	Description
Acceptability	The perception among implementation stakeholders that a specific intervention or practice is agreeable or satisfactory (or aspects of e.g. content or complexity) within a defined setting
Adoption	The intentional ‘uptake’ or initial decision to undertake an evidence-based intervention or practice
Appropriateness	The perceived fit, relevance or compatibility of the intervention or practice for a given setting, provider or consumer and/or perceived fit to address a particular issue
Cost	The cost of the intervention, the implementation strategies and any additional costs required to support its implementation
Feasibility	The extent to which a new intervention or practice can be successfully used or carried out within a given service or setting
Fidelity—Adherence	The degree to which the intervention content is implemented as per protocol/manual
Fidelity—Dose	The amount of the intervention delivered to or received by participants
Fidelity—Quality of delivery	The effectiveness of intervention strategies for delivery
Fidelity—Participant responsiveness	The level of engagement in and receptiveness to the intervention by participants
Fidelity—Program Differentiation	Whether essential elements of the intervention implementation distinguish treatment from comparison groups when evaluating its efficacy
Penetration	The ‘reach’ or integration of an intervention or practice within a setting and its subsystems
Sustainability	The extent to which a newly implemented intervention or practice is maintained or institutionalised within a setting’s ongoing operations

Definitions used to determine the type of effectiveness-implementation trial were adapted from Curran et al. (2022); Type 1: Implementation outcomes collected and reported but not part of relational health intervention effectiveness analysis (i.e., fidelity checks); Type 2: Implementation outcomes collected and used in relational health intervention effectiveness analysis (i.e., focus both on intervention and implementation impacts); Type 3: Intervention tested is an implementation strategy, with implementation effectiveness analysis conducted and relational health outcomes collected and reported. The identification of implementation strategies was guided by compilations by Powell et al. (2015) and Leeman et al. (2017).

No follow-up with authors regarding additional implementation-related publications was conducted due to potentially inconsistent responses and subsequent bias in the analysis. Only the RCTs sourced through the scoping review were included and no reporting on the effectiveness or the methodological quality of the RCTs was conducted.

### Data Screening and Extraction

The original scoping review by Allen et al. (2026) of relational health interventions identified 125 papers for inclusion. As two papers were addendums to previously published papers and did not provide any further details on the intervention’s implementation, these were excluded (Heinrichs et al., 2017; Prinz et al., 2016). A total of 123 discrete studies were retained for full-text screening and data extraction (See Table 2 for data extracted). The extraction strategy was piloted on five intervention studies and refined throughout the extraction process. Implementation-related data were extracted by three authors (JSK, AG and CA) trained in implementation science concepts. Approximately 50% of the studies were checked by the first author (NP), an experienced implementation scientist, to ensure the consistent application of concepts during data extraction. When implementation study features were unclear, team members consulted the authorship team. This process led to greater consistency and minor changes to the review process (e.g., clarification of terms). Most difficulties stemmed from the inconsistent use of terminology across the papers given the field of implementation science’s relative infancy. Some variables extracted during the original scoping review were also included in the analysis (age group; relational health dependent variable; intervention type and setting; socio-ecological level; and population or community-based intervention).

**Table 2:** Data extraction variables and response categories

Variables	Response categories
Inclusion of implementation by type of effectiveness-implementation trial (Curran et al., 2022)	Type 1 trial—Implementation outcomes reported but not used in intervention effectiveness analysis Type 2 trial—Implementation outcomes reported and used in intervention effectiveness analysis Type 3 trial—Intervention tested is an implementation strategy, with implementation effectiveness analysis conducted and relational health outcomes reported Implementation discussed only Not included
Implementation and fidelity outcomes Carroll et al. (2007); Gearing et al. (2011); Proctor et al. (2011)	Acceptability Adoption Appropriateness Cost Feasibility Fidelity – adherence Fidelity – dose Fidelity – quality of delivery Fidelity – participant responsiveness Fidelity – program differentiation Penetration Sustainability
Implementation data collection methods	Participant self-report survey Interview Facilitator self-report checklist/log Independent observer Study records Does not state
Implementation measures	Validated measure Measure specific to study Does not state
Implementation theories/models/ frameworks	Open-ended response
Age group	Early childhood Childhood Adolescence Childhood and Adolescence Young adult
Relational health outcomes	Aggression and violence Aggressive and disruptive behaviour Child abuse and maltreatment Community cohesion and social capital Family relationships Intimate partner relationship Intimate partner violence Parent-school relationship Parenting Peer relationships Protective factors Risky sexual behaviours and attitudes School climate School engagement Social competence Teacher–child relationship
Socio-ecological level	Microsystem Exosystem Microsystem & mesosystem Microsystem & exosystem Microsystem, mesosystem & exosystem
Intervention setting	Early childcare School School and home/community Community Primary care Online Laboratory Laboratory and online
Intervention type	Classroom-based curriculum Community prevention system Family-based multicomponent Group sessions for children Multicomponent support for parents Parenting Peer-led intervention School-based multicomponent Teacher training Welfare/policy reforms Whole-school program Primary health-based
Population or community-based	Multiple geographic areas: Yes/No

### Data Analysis

Descriptive analyses of the extracted data were undertaken using SPSS Version 27. A narrative synthesis of the study findings was conducted (Ryan & Cochrane Consumers and Communication Review Group, 2013).

## Results

### General characteristics of included studies and interventions

The general characteristics of the 85 interventions identified in the 123 studies can be found in Table 2 of Allen and colleagues’ scoping review (Allen et al., 2026). In brief, over 90% of the interventions targeted children and adolescents aged 4–18 years, with only three interventions targeting young adults (19–24 years). Most of the studies were conducted in the US (58%) and Western Europe and Scandinavia (20%). Of the 35 interventions evaluated outside of the US, seven tested US-developed interventions in a different country. Around 50% of the RCT studies captured in this review were published in the last 10 years, however, some of the interventions were long running (some initiated 15–20 years ago) and widely published. Seventy-five percent of the interventions were described in one study only, 15% of the interventions were described in two studies and 9% of the interventions were described in three to seven studies (Good Behaviour Game, Prosper, Triple P, Communities that Care, Coping Power, Positive Action, KiVa Anti-bullying Program, School-Wide Positive Behavioural Interventions and Supports and The GREAT Student Curriculum and GREAT Teacher Program).

Twelve categories were used to describe the type of intervention (See Table 2), with the most common being classroom-based curriculum (35%, 43 studies), parenting interventions (16%, 20 studies), school-based multicomponent (10%, 12 studies), community prevention system (10%, 12 studies) and family-based multicomponent (5%, 5 studies). The most common relational health outcomes measured (across 16 categories; See Table 2) were peer relationships (19%, 23 studies), social competence (15%, 18 studies), parenting (14%, 17 studies), aggression and violence (11%, 13 studies) and risky sexual behaviours and attitudes (11%, 13 studies). While interventions can be delivered at multiple levels of a child’s social ecology, most of the studies (98%) focussed solely on the microsystem (e.g., immediate family and caregivers, teachers, peers), 17% included the mesosystem level (e.g., interactions between microsystems such as parent-teacher relationships) and 15% the exosystem level (e.g., wider social services and policies).

### Implementation Characteristics of Included Studies and Interventions

This secondary analysis assessed RCTs according to a range of implementation-related characteristics (See Tables 3 and 4).

**Table 3:** Intervention characteristics of included studies by trial type (123 studies; 85 interventions)

Trial Type	Age-group % of studies	Ecological level % of studies	Setting % of studies	Intervention type % of studies	Relational health outcomes^*^% of outcomes	Studies
Type 3(Evaluation of the effectiveness of an implementation strategy. Minor reporting of relational health outcomes) 5 studies; 4 interventions	Adolescence 80% Childhood 20%	Microsystem 80% Microsystem, Mesosystem & Exosystem 20%	School 60% School and home/community 20% Online 20%	Classroom-based curriculum 40% Whole-school program 20% Parenting 20% Community prevention system 20%	Teacher–child relationships 53% Parenting 12% Family relationships 9% Peer relationships 9% Social competence 9% Aggressive and disruptive behaviour 3% Community cohesion and social capital 3% School climate 3%	^1–5^
Type 2 (Evaluation of the effectiveness of both implementation and relational health outcomes) 16 studies; 16 interventions	Childhood 57% Adolescence 25% Early Childhood 6% Childhood and Adolescence 6% Young Adult 6%	Microsystem 75% Microsystem & Mesosystem 19% Microsystem & Exosystem 6%	School 50% Community 25% Early childcare 6% School and home/community 6% Primary care 6% Online and Laboratory 6%	Parenting 37.5% School-based multicomponent 19% Classroom-based curriculum 12.5% Group sessions for children 12.5% Whole-school program 12.5% Primary health-based 6%	Risky sexual behaviours and attitudes 30% Parenting 28% Intimate partner violence 11.5% Peer relationships 10% Aggression and violence 8% Social competence 7% Family relationships 4.5% Aggressive and disruptive behaviour 1%	^6–21^
Type 1 (Evaluation of the effectiveness of relational health outcomes. Minor reporting on implementation) 64 studies; 52 interventions	Childhood 60% Adolescence 29% Early Childhood 8% Childhood and Adolescence 3%	Microsystem 84% Microsystem & Mesosystem 8% Microsystem, Mesosystem & Exosystem 6% Exosystem 2%	School 67% Community 14% Early childcare 10% School and home/community 8% Laboratory 2%	Classroom-based curriculum 40% Parenting 18% Whole-school program 11% School-based multicomponent 8% Family-based multicomponent 8% Community prevention system 5% Teacher training 3% Group sessions for children 3% Welfare/policy reforms 2% Peer-led intervention 2%	Parenting 22% Social competence 22% Peer relationships 14% Aggression and violence 10% Risky sexual behaviours and attitudes 10% Aggressive and disruptive behaviour 6% Family relationships 5% Child abuse and maltreatment 3% School climate 3% Teacher–child relationships 2% School engagement 1% Intimate partner violence 1% Parent-school relationship 1%	^22–85^
Discussion only 26 studies; 21 interventions	Adolescence 56% Childhood 33% Young Adult 7% Early Childhood 4%	Microsystem 77% Microsystem, Mesosystem & Exosystem 19% Microsystem & Exosystem 4%	School 59% School and home/community 19% Community 15% Early childcare 7%	Classroom-based curriculum 38% Community prevention system 27% Whole-school program 15% Multicomponent support for parents 4% Parenting 4% School-based multicomponent 4% Teacher training 4% Primary health-based 4%	Intimate partner violence 22% Peer relationships 19% School climate 9% Aggressive and disruptive behaviour 8.5% Social competence 7.5% Teacher–child relationships 7.5% Aggression and violence 7.5% Parenting 5% Child abuse and maltreatment 4% Community cohesion and social capital 3% School engagement 3% Intimate partner relationship 2% Family relationships 1% Risky sexual behaviours and attitudes 1%	^86–111^
Not addressed 12 studies; 9 interventions	Adolescence 67% Childhood 33%	Microsystem 67% Microsystem, Mesosystem & Exosystem 17% Microsystem & Exosystem 8% Exosystem 8%	School 75% School and home/community 25%	Classroom-based curriculum 25% School-based multicomponent 25% Community prevention system 17% Whole-school program 17% Family-based multicomponent 8% Group sessions for children 8%	Intimate partner violence 56% Peer relationships 19% Risky sexual behaviours and attitudes 12% School climate 5% Aggression and violence 4% Family relationships 2% Protective factors 2%	^112–123^

^*^More than one relational health outcome possible per study

^1^(Calam et al., 2008); ^2^(Chirimwami & Van Ryzin, 2024); ^3^(Debnam et al., 2024); ^4^(Feinberg et al., 2010); ^5^(Low & Van Ryzin, 2024); ^6^(Arango et al., 2024); ^7^(Botvin et al., 2006); ^8^(Cina et al., 2024); ^9^(Coulter et al., 2024); ^10^(Dawson-McClure et al., 2015); ^11^(Eisner et al., 2012); ^12^(Fabrizio et al., 2014); ^13^(Kiviruusu et al., 2016); ^14^(Klocek et al., 2024); ^15^(Kosterman et al., 2001); ^16^(Linhares et al., 2022); ^17^(Mennicke et al., 2021); ^18^(Rincón et al., 2018); ^19^(Stanton et al., 1996); ^20^(The Multisite Violence Prevention Project, 2014); ^21^(Waidler et al., 2022); ^22^(Allen et al., 2021); ^23^(Basen-Engquist et al., 2001); ^24^(Berger et al., 2018); ^25^(Bierman et al., 2014); ^26^(Blair et al., 2024); ^27^(Bradshaw et al., 2012); ^28^(Conduct Problems Prevention Research Group, 2010); ^29^(Chu et al., 2015); ^30^(Chuang et al., 2020); ^31^(Cova et al., 2019); ^32^(Crooks et al., 2011); ^33^(Farrell et al., 2001); ^34^(Fein & Lee, 2003); ^35^(Fishbein et al., 2016); ^36^(Flannery et al., 2003); ^37^(Flay et al., 2004); ^38^(Fonagy et al., 2009); ^39^(Foshee et al., 2014); ^40^(Francis et al., 2016); ^41^(Haar et al., 2021); ^42^(Hahlweg et al., 2010); ^43^(Heinrichs et al., 2014); ^44^(Ialongo et al., 2019); ^45^(Jensen et al., 2014); ^46^(Jones et al., 2010); ^47^(Jones et al., 2011); ^48^(Juvonen et al., 2016); ^49^(Kim et al., 2023); ^50^(Kratochwill et al., 2009); ^51^(Leflot et al., 2013); ^52^(Letourneau et al., 2022); ^53^(Lewis et al., 2016); ^54^(Lochman & Wells, 2002); ^55^(Low et al., 2023); ^56^(Mageau et al., 2022); ^57^(Muratori et al., 2015); ^58^(Muratori et al., 2016); ^59^(Muratori et al., 2019); ^60^(Muratori et al., 2020); ^61^(Murry et al., 2007); ^62^(Osgood et al., 2013); ^63^(Pontes & Brino, 2022); ^64^(Prinz et al., 2009); ^65^(Puffer et al., 2015); ^66^(Redmond et al., 2009); ^67^(Reid et al., 1999); ^68^(Rotz et al., 2018); ^69^(Sanders et al., 2008); ^70^(Sim et al., 2025); ^71^(Snyder et al., 2013); ^72^(Spoth et al., 2014); ^73^(Spoth et al., 2017); ^74^(Stefan et al., 2023); ^75^(Streimann et al., 2020); ^76^(Sullivan et al., 2017); ^77^(The Multisite Violence Prevention Project, 2009); ^78^(The Multisite Violence Prevention Project, 2008); ^79^(van Lier et al., 2011); ^80^(Volkaert et al., 2021); ^81^(Waasdorp et al., 2012); ^82^(Webster-Stratton et al., 2008); ^83^(Wilson et al., 2012); ^84^(Wong et al., 2014); ^85^(Zhai et al., 2015); ^86^(Aber et al., 2017); ^87^(Bai et al., 2025); ^88^(Chikwari et al., 2025); ^89^(Chilenski et al., 2014); ^90^(Coker et al., 2020); ^91^(DeGue et al., 2021); ^92^(Dowling et al., 2019); ^93^(Edwards et al., 2019); ^94^(Espelage et al., 2013); ^95^(Havighurst et al., 2022); ^96^(Hawkins et al., 2012); ^97^(Hull et al., 2021); ^98^(Kärnä et al., 2011); ^99^(Kellam et al., 2014); ^100^(Kubiszewski et al., 2023); ^101^(LoBraico et al., 2022); ^102^(Mogro-Wilson et al., 2020); ^103^(Noll et al., 2025)^c^; ^104^(Oesterle et al., 2018); ^105^(Petras et al., 2008)^a^; ^106^(Reedtz et al., 2011); ^107^(Tevendale et al., 2024); ^108^(Thulin et al., 2022); ^109^(van Lier et al., 2005)^a^; ^110^(Vazsonyi et al., 2004); ^111^(Wolfe et al., 2009); ^112^(Brincks et al., 2023); ^113^(Mertens et al., 2022); ^114^(Newcomer et al., 2016)^a^; ^115^(Nocentini et al., 2019); ^116^(Oesterle et al., 2014)^c^; ^117^(Smokowski et al., 2020); ^118^(Ssewamala et al., 2021); ^119^(Ssewamala et al., 2023); ^120^(Taylor et al., 2015); ^121^(Taylor et al., 2017a); ^122^(Toumbourou et al., 2019)^c^; ^123^(Vuijk et al., 2007)

**Table 4:** Intervention characteristics of included studies by implementation outcome (123 studies; 85 interventions)

Implementation outcome^*^	Age group^**^	Ecological level^**^	Setting^**^	Intervention type^**^	Data collection methods^**^	Studies
Acceptability Measured 10 times across 8 studies; 6 interventions	Childhood 70% Early childhood 20% Adolescence 10%	Microsystem 80% Microsystem & Mesosystem 20%	School 60% Early childcare 20% Community 20%	Parenting 50% School-based multicomponent 20% Whole-school program 14% Classroom-based curriculum 10%	Participant self-report survey 90% Interview 10%	^1–8^
Feasibility Measured once across 1 study; 1 intervention	Adolescence 100%	Microsystem 100%	School 100%	Group sessions for children 100%	Participant self-report survey 100%	^9^
Adherence to content Measured 48 times across 48 studies; 36 interventions	Childhood 58% Adolescence 29% Early childhood 10% Childhood and Adolescence 2%	Microsystem 77% Microsystem & Mesosystem 15% Microsystem, Mesosystem & Exosystem 9%	School 65% School and Home/Community 13% Community 12% Early childcare 10%	Classroom-based curriculum 33% Parenting 21% School-based multicomponent 12% Family-based multicomponent 11% Whole-school program 10% Community prevention system 7% Group sessions for children 2% Teacher training 4%	Independent observer 56% Facilitator self-report checklist/log 33% Doesn’t state 9% Study records 2%	^3, 5–9, 10–51^
Dose Measured 73 times across 57 studies; 50 interventions	Childhood 64% Adolescence 26% Early childhood 5% Childhood and Adolescence 3% Young Adulthood 1%	Microsystem 88% Microsystem & Mesosystem 8% Microsystem, Mesosystem & Exosystem 2% Microsystem & Exosystem 1% Exosystem 1%	School 59% Community 21% Early childcare 7% School and Home/Community 6% Primary Care 1% Laboratory 1% Online 1% Laboratory and Online 1%	Classroom-based curriculum 38% Parenting 26% Family-based multicomponent 10% School-based multicomponent 8% Whole-school program 8% Group sessions for children 4% Peer-led intervention 1% Welfare/policy reforms 1% Primary health-based 1% Community prevention system 1%	Study records 35% Facilitator self-report checklist/log 33% Participant self-report survey 13% Independent observer 12% Doesn’t state 7%	^1–6, 8, 16–18, 21–23, 25–26, 28–31, 36, 39, 43, 46–47, 52–85^
Quality of delivery Measured 11 times across 10 studies; 10 interventions	Early childhood 9% Childhood 73% Adolescence 18%	Microsystem 91% Microsystem & Mesosystem 9%	School 64% Early childcare 27% Community 9%	Classroom-based curriculum 27% School-based multicomponent 27% Whole-school program 27% Parenting 19%	Independent observer 64% Participant self-report survey 18% Interview 9% Facilitator self-report checklist/log 9%	^1–3, 16, 22, 44, 53–54, 60, 82^
Participant responsiveness Measured 6 times across 6 studies; 5 interventions	Childhood 50% Adolescence 50%	Microsystem 100%	School 67% Early childcare 16.5% School and home/community 16.5%	Classroom-based curriculum 33% Family-based multicomponent 17% School-based multicomponent 17% Whole-school program 17% Parenting 17%	Facilitator self-report checklist/log 33% Independent observer 33% Study records 17% Doesn’t state 17%	^41, 44, 45–46, 51, 82^
Cost Measured 4 times across 4 studies; 3 interventions	Childhood 100%	Microsystem 75% Microsystem & Mesosystem 25%	School 100%	Classroom-based curriculum 75% Family-based multicomponent 25%	Study records 50% Doesn’t state 50%	^26, 68–70^

Five implementation outcomes (adoption; appropriateness; fidelity—differentiation; penetration; sustainability) are not included as no studies measured these.

^**^Percentages refer to proportion of measured implementation outcomes in each category (percentages may not equal 100 due to rounding).

^1^(Debnam et al., 2024); ^2^(Eisner et al., 2012); ^3^(Fabrizio et al., 2014); ^4^(Flannery et al., 2003); ^5^(Hahlweg et al., 2010); ^6^(Heinrichs et al., 2014); ^7^(Lewis et al., 2016); ^8^(Reid et al., 1999); ^9^(Volkaert et al., 2021); ^10^(Arango et al., 2024); ^11^(Berger et al., 2018); ^12^(Bradshaw et al., 2012); ^13^(Chu et al., 2015); ^14^(Chuang et al., 2020); ^15^(Cova et al., 2019); ^16^(Dawson-McClure et al., 2015); ^17^(Farrell et al., 2001); ^18^(Fishbein et al., 2016); ^19^(Flay et al., 2004); ^20^(Fonagy et al., 2009); ^21^(Haar et al., 2021); ^22^(Ialongo et al., 2019); ^23^(Jensen et al., 2014); ^24^(Juvonen et al., 2016); ^25^(Kosterman et al., 2001); ^26^(Kratochwill et al., 2009); ^27^(Leflot et al., 2013); ^28^(Letourneau et al., 2022); ^29^(Lochman & Wells, 2002); ^30^(Low et al., 2023); ^31^(Mageau et al., 2022); ^32^(Muratori et al., 2015); ^33^(Muratori et al., 2016); ^34^(Muratori et al., 2019); ^35^(Muratori et al., 2020); ^36^(Murry et al., 2007); ^37^(Osgood et al., 2013); ^38^(Redmond et al., 2009); ^39^(Rincón et al., 2018); ^40^(Snyder et al., 2013); ^41^(Spoth et al., 2014); ^42^(Spoth et al., 2017); ^43^(Streimann et al., 2020); ^44^(Sullivan et al., 2017); ^45^(The Multisite Violence Prevention Project, 2008); ^46^(The Multisite Violence Prevention Project, 2009); ^47^(The Multisite Violence Prevention Project, 2014); ^48^(Waasdorp et al., 2012); ^49^(Webster-Stratton et al., 2008); ^50^(Wilson et al., 2012); ^51^(Wong et al., 2014); ^52^(Allen et al., 2021); ^52^(Basen-Engquist et al., 2001); ^53^(Bierman et al., 2014); ^54^(Blair et al., 2024); ^55^(Botvin et al., 2006); ^57^(Calam et al., 2008); ^58^(Chirimwami & Van Ryzin, 2024); ^59^(Cina et al., 2024); ^60^(Conduct Problems Prevention Research Group, 2010); ^61^(Coulter et al., 2024); ^62^(Crooks et al., 2011); ^63^ (Fein & Lee, 2003); ^65^(Feinberg et al., 2010); ^66^(Foshee et al., 2014); ^67^(Francis et al., 2016); ^68^(Jones et al., 2010); ^69^(Jones et al., 2011); ^70^(Kim et al., 2023); ^71^(Kiviruusu et al., 2016); ^72^(Klocek et al., 2024); ^73^(Linhares et al., 2022); ^74^(Mennicke et al., 2021); ^75^(Pontes & Brino, 2022); ^76^(Prinz et al., 2009); ^77^(Puffer et al., 2015); ^78^(Rotz et al., 2018); ^79^(Sanders et al., 2008); ^80^(Sim et al., 2025); ^81^(Stanton et al., 1996); ^82^(Stefan et al., 2023); ^83^(van Lier et al., 2011); ^84^(Waidler et al., 2022); ^85^(Zhai et al., 2015).

#### Relational Health and Implementation Outcomes

Of the 111 studies that at least discussed implementation outcomes, the most common primary relational health outcomes assessed were social competence (16%; 18 studies), peer relationships (16%; 18 studies) and parenting (15%; 17 studies). Notably, there was an absence or low number of studies that included implementation outcomes and addressed specific relational health outcomes, such as protective factors (0 studies), intimate partner relationship (2 studies), child abuse and maltreatment (3 studies), school engagement (2 studies), teacher-student relationship (2 studies), parent-school relationship (1 studies) and community cohesion and social capital (1 studies).

#### Inclusion of Implementation Outcomes in Intervention Trials

The full text review showed that implementation outcomes were rarely integrated into universal relational health intervention trials (See Table 3). In 83% of studies, implementation outcomes appeared only as a minor analyses or brief reporting (Type 1 trial: 52%; 64 studies), were mentioned solely in the discussion (21%; 26 studies), or were not included at all (10%; 12 studies). Only 17% of studies incorporated implementation outcomes within a hybrid effectiveness-implementation design, either as a Type 2 (13%; 16 studies) or Type 3 (4%; 5 studies) trial.

#### Type 2 and 3 Effectiveness-Implementation Trials

All 85 interventions published across 123 trials aimed to improve relational health outcomes for children and families as part of the original scoping review criteria. However, of the 111 studies that addressed implementation, only 5 studies (4%; 4 interventions) tested an implementation strategy and hence included implementation outcomes as the primary impact analysis as a Type 3 effectiveness-implementation study. A further 13% were Type 2 studies (16 studies; 16 interventions) that included implementation outcomes as a dual objective and analysis (along with relational health outcomes) (See Table 3).

A significant proportion of these Type 2 and 3 studies focussed on the microsystem (75% and 80% respectively) and were conducted within the school setting (50% and 60% respectively). Most of the Type 3 studies were conducted with adolescents (80%) whereas more Type 2 studies were conducted with children (4–12 years; 57%). Parenting interventions were the most common type of intervention for Type 2 studies (37.5%) whereas the most common type of intervention in Type 3 studies was classroom-based curriculum (40%). Improved teacher–child relationships was the most common relational health outcome measured in Type 3 studies (53%; 18 studies). Whereas for Type 2 studies, risky and sexual behaviours and attitudes (30%; 27 studies) and parenting (28%; 25 studies) were the most common.

#### Type 1 Effectiveness-Implementation Trials

Over a half of the studies (52%; 64 studies; 52 (59%) interventions) included implementation outcomes as a minor analysis or reporting that were not part of the study aims or impact analysis (Type 1 study) (See Table 3). Of the Type 1 studies, 60% (38 studies) targeted children, 29% adolescents (19 studies) and 3% (2 studies) targeted both children and adolescents together. The most common settings were school (67%; 43 studies) and community-based (14%; 9 studies). Classroom-based curriculum was the most common type of intervention (40%; 26 studies). Social competence (22%; 65 studies), parenting (22%; 66 studies) and peer relationships (14%; 41 studies) were the most common relational health outcomes measured in Type 1 studies.

#### Implementation Outcomes Discussed Only

Twenty-one percent of studies (26 studies; 21 (26%) interventions) addressed implementation issues in their discussion only with no analysis or reporting (See Table 3). In contrast to the Type 1 studies, a higher proportion of studies that only discussed implementation involved adolescents (56%; 14 studies) and included two of the three studies conducted with young adults. Similar to Type 1 studies, the primary setting was school (59%; 15 studies) and school and home/community (19%; 5 studies). The most common relational health outcomes included intimate partner violence (20%; 24 studies) and peer relationships (16.5%; 20 studies). Whilst classroom-based curriculum (38%; 10 studies) and community prevention system interventions (27%; 7 studies) were most common, whole-school interventions (15%; 4 studies) were also featured. For those studies that mentioned implementation in their discussion section only, the reference to implementation was usually brief and recommended further research, including implementation measurement or focus to improve intervention effectiveness and translation efforts.

#### Implementation Outcomes not Included

Twelve out of the 123 studies (10%; 9 (10%) interventions) did not include any implementation outcomes in their study design, analysis or discussion (See Table 3). Approximately two-thirds of the RCTs that did not address implementation, sampled adolescents (67%; 8 studies) and focussed on the microsystem (67%; 8 studies). All 12 studies were conducted in the school and school and home/community setting and therefore, two-thirds of the interventions were types of school-based interventions (e.g. classroom-based curriculum, whole-school or multicomponent) and focused on issues such as peer relationships, intimate partner violence and risky sexual behaviours and attitudes.

#### Implementation Theories and Frameworks

No studies described an established implementation theory or framework (e.g., CFIR, RE-AIM or Diffusion of Innovations Theory) as part of their study aims, methods or analysis (Birken et al., 2017; Wang et al., 2023). If a theory was mentioned, it was related to the development of intervention content or individual behaviour change mechanisms (e.g. Social Development Model), not in relation to implementation strategies or research methodology. Some studies briefly referenced their intervention manual or program model (e.g. School-wide Positive Behavioural Interventions and Supports (SWPBIS), CASEL Framework, Good Behaviour Game Framework or Communities That Care Model) that may have addressed their delivery or implementation plans, however, these details were not provided within the included review publications.

#### Implementation Outcomes

**Fig. 1:**
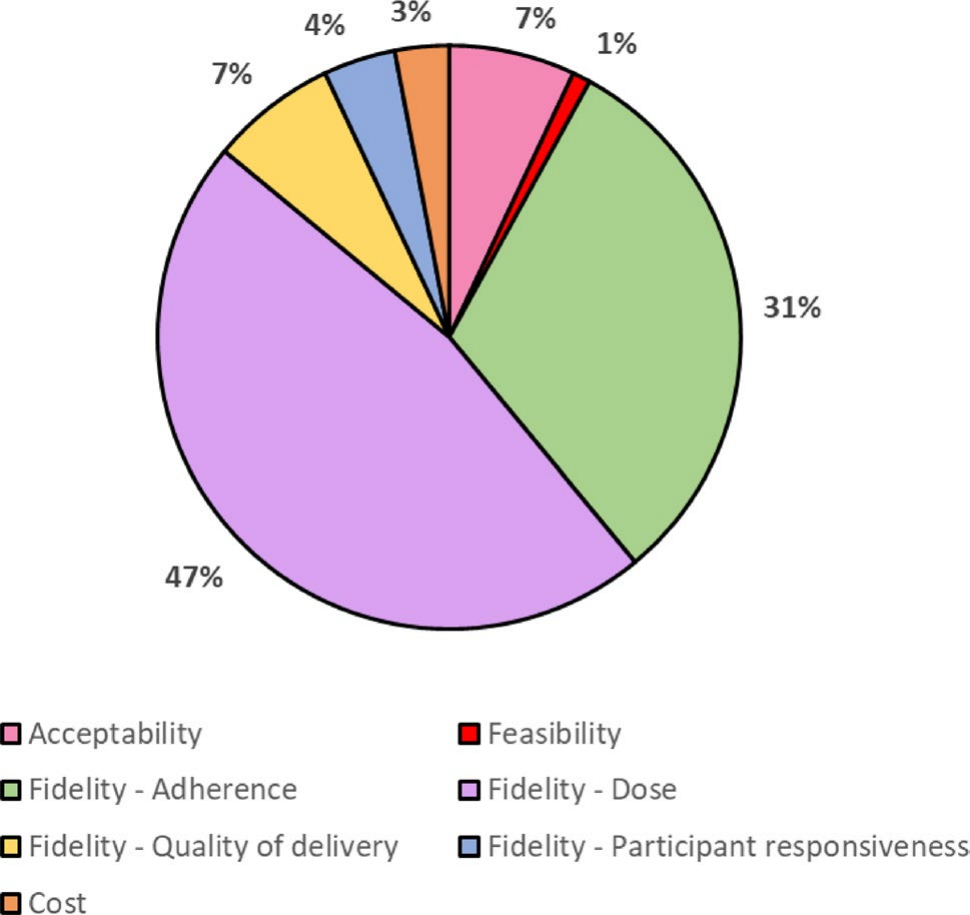
Percentage of studies reporting implementation outcomes (n = 123). *Note*: Implementation outcomes are not shown if not reported by any studies

The 123 evaluation trials included in this review were assessed for their inclusion of 12 implementation outcomes commonly measured in the implementation science literature (see Table 1 for definitions). There was no mention of five of the twelve implementation outcomes (42%), namely adoption, appropriateness, fidelity- differentiation, penetration and sustainability. The most frequently included implementation outcomes were fidelity measures of dose (48%; 57 studies; 50 interventions) and adherence (31%; 48 studies; 36 interventions), together totalling 79% of the included implementation outcomes (See Table 4). For the remaining, both acceptability (8 studies; 6 interventions) and quality of delivery (10 studies; 10 interventions) accounted for 7% each, participant responsiveness 4% (6 studies; 5 interventions), cost 3% (4 studies; 3 interventions) and feasibility 1% (1 study; 1 intervention) (See Fig. 1).

A significant proportion of the intervention studies’ that included implementation outcomes targeted childhood (63%) and, to a lesser extent, adolescence (26%) (See Table 4). Across all the implementation outcomes measured, the most common intervention setting was school-based (range 59% to 100%) followed by early childhood (range 7% to 27%). Hence the most common type of intervention was classroom-based curriculum accounting for 35% of the implementation outcomes reported. followed by parenting interventions (24%). Across all studies that included implementation outcome measures, the majority were collected within the ecological microsystem (range 75–100%) with very few measured for the meso- or exosystems. Studies measuring outcomes of dose were more likely to use facilitator checklists (33%) and study records (35%) to collect their data, whereas studies measuring adherence to intervention content were more likely to use independent observers (56%). For implementation outcomes targeting participant feedback, like acceptability and feasibility, most studies used participant self-report surveys.

#### Implementation Data Collection Methods and Measures

Almost two-thirds of the studies that measured implementation outcomes collected these data using facilitator self-report checklists (28%; 36 studies) or independent observers (29%; 38 studies) (See Online Resource 1). An additional 19% (25 studies) used study records to measure implementation outcomes (e.g., attendance records) or participant self-report surveys (14%; 19 studies). A further 9% (13 studies) provided no detail about their data collection methods and 1% (2 studies) used interviews. Nearly a quarter (23%) of the studies provided no details of the implementation related measures used and 77% of the studies reported developing their own study-specific implementation measures (or were recorded as such if they used study records like attendance or participation rates). None of the studies cited standardised implementation outcome measures described in the implementation science literature (Mettert et al., 2020).

#### Population and Community Level Intervention Trials

Nineteen studies (9 interventions; see Online Resource 2) were considered population or community-based trials as they were conducted across multiple geographical areas (e.g., countries, state, districts, local government areas). Of these, one study was a Type 3 effectiveness-implementation trial that examined intervention effectiveness for high dose participants (Communities that Care), and one was a Type 2 trial (Ujana Salama) that examined effectiveness by attendance and engagement in program components. Seven studies were a Type 1 trial design and eight studies mentioned implementation only in their discussion. Two population trial studies did not mention implementation outcomes. Of the nine (47%) population or community-based trials that included implementation measurement, the most common implementation outcomes measured were dose (6 studies; e.g., amount of training delivered or received) and adherence to intervention content (4 studies), The types of interventions varied from community prevention systems approaches (e.g., PROSPER and Communities That Care) to classroom-based curriculum (e.g., PATHS and Learning to Read in a Healing Classroom) and parenting interventions (e.g., Triple P) and targeted a range of relational health outcomes.

## Discussion

Given the effectiveness of interventions is influenced by the way they are implemented, measurement of their implementation is critical. Durlak and DuPre (2008) state: “Implementation is when a programme becomes what it is” (p.378). This paper aimed to understand the extent to which RCTs of universal relational health interventions also assessed implementation outcomes to support the intervention’s effectiveness.

This review found that only twenty-one studies assessed implementation outcomes as a significant focus along with relational health outcomes, with most of the studies reporting implementation outcomes separate to the intervention’s impact, in their discussion only or not at all. The most frequently reported implementation outcomes were fidelity measures of dose and adherence and almost a half of the twelve pre-defined implementation outcomes were not addressed. No studies described an implementation-related theory or framework and all used non-standardised implementation measures developed specifically for their study. This finding has important implications for the future scaling of these interventions and their effectiveness when used by practitioners in clinical and community settings.

### Concurrent Measurement of Relational Health and Implementation Outcomes

A Type 2 and 3 hybrid study which intentionally and concurrently assesses an intervention’s effectiveness and implementation to explain intervention effects, was used by 21 studies (E.g., Botvin et al. (2006); Feinberg et al. (2010); Rincón et al. (2018); The Multisite Violence Prevention Project (2014)). While fewer Type 3 hybrid studies that tested a discrete implementation strategy were expected, it was surprising how few Type 2 hybrid studies were found that measure both the intervention’s effectiveness and implementation to explain intervention effects. Nonetheless, these findings are consistent with other research suggesting the interaction between the implementation process and intervention effectiveness is rarely captured in efficacy and effectiveness studies (Coldwell & Moore, 2024; O'Donnell, 2008; Wolfenden et al., 2019). It is imperative that relational health intervention research considers Type 2 hybrid study designs that assess implementation and its effects on intervention outcomes to inform their replicability and scalability to practice.

Green (2014) argues that an RCT without implementation data decontextualises most of the evidence it produces, whereas a hybrid design blends ‘rigor and reality’ by providing an opportunity to expand external validity while maintaining internal validity. A hybrid study can provide a clearer understanding of whether the hypothesised mechanisms caused the effects, and possibly how and for whom, while also helping to identify and address barriers and facilitators to the uptake of the relational health intervention (Bernet et al., 2013; Curran et al., 2012; Wolfenden et al., 2021). Gaffney et al. (2019) for example, found that variable levels of implementation for each intervention component may explain variability in intervention outcomes—i.e., of those relational health interventions found to be effective, do they need to be replicated in full or could some components or processes be modified? Most studies in this review were Type 1 hybrid studies which is a standard RCT design that reports implementation outcomes, such as fidelity, but these implementation outcomes are not part of the intervention impact analyses. This suggests there is an urgent need to strengthen trial designs to capture implementation outcomes and processes. Alternative trial designs could also be considered, such as stepped wedge, that stagger intervention implementation to different sites to overcome practical, ethical and scientific concerns, rather than traditional parallel-group designs (Simon et al., 2025).

### Using Implementation Data to Increase Validity of Intervention Effectiveness

Most of the Type 2 hybrid studies in this review demonstrated robust fidelity analyses to assess whether the intervention was being delivered with the intended level of intensity, quality, and adherence (Coulter et al., 2024; Stains & Vickrey, 2017; Toomey et al., 2020). These studies were used to understand how or why relational outcomes were influenced by the degree of implementation fidelity by including an implementation variable (i.e., level of implementation score) as a covariate in models testing intervention effects or conducting a separate analyses with the sample who received more than a half of the intervention (high fidelity). These studies found greater effects when the intervention was implemented with higher fidelity (e.g., high-quality implementation of PATHS) (Botvin et al., 2006; Kosterman et al., 2001; Waidler et al., 2022). This approach, called ‘treatment-on-the-treatment-one’ (TOT) or ‘per-protocol’ analyses, focusses on participants who received the intervention as intended (Linhares et al., 2022; Tripepi et al., 2020; Waidler et al., 2022). Humphrey et al. (2016) argue that an RCT can only demonstrate whether an impact occurred if it is able to link the dose of the intervention to how or why the intended outcomes changed.

In contrast to the ‘per-protocol’ analyses, most RCTs in this review used an Intention-To-Treat (ITT) approach (Type 1 studies). This approach assumes that intervention effects are consistent across all participants and therefore provides a conservative estimate of effects in real-life scenarios at the population level, by including all participants, regardless of how well they received the intervention (DeGue et al., 2021; Jensen et al., 2014; Leflot et al., 2013; Mageau et al., 2022). However, Eisner et al. (2012) state that ITT analyses are of limited value, due to diluted effects, and should only be used with those who receive the full intervention. Whilst they used a more innovative method of propensity score matching, a more common method is a dose–response analysis assessing the relationship between relational health outcomes and a sample receiving a ‘full dose’ of the intervention, similarly offering insights into the effectiveness of the intervention (Dawson-McClure et al., 2015).

Some may argue that an ITT approach is sufficient in an efficacy trial, where conditions are tightly controlled and the intervention components limited to assess initial impact, however, effectiveness trials account for the real-life complexities of implementing interventions into existing community and service systems and therefore must evaluate implementation outcomes. Regardless of the stage of research, all RCT trials should assess implementation factors and outcomes and conduct both ITT and TOT analyses to inform their scaling into community and clinical settings and increase impacts for children and families. Conducting both an ITT and TOT analyses within trials will also reduce biases and balance internal and external validity of the findings, as demonstrated by Linhares et al. (2022).

### Measurable Outcomes and Assessment of the Process and Quality of how Interventions are Implemented

While it may not be possible to fully understand how variations in relational health intervention delivery affected outcomes, this review found that implementation outcomes overall were inadequately measured and reported. Almost a half of the twelve pre-defined implementation outcomes were not addressed (adoption, appropriateness, differentiation, penetration and sustainability). Fidelity measures of dose and adherence were the most common implementation outcomes measured, with a few studies each reporting intervention acceptability, quality of delivery, participant responsiveness, cost and feasibility. It is likely that the settings of these relational intervention studies enabled researchers to collect fidelity and adherence data more easily, as many targeted children in schools and focused on ecological microsystems. Of note, none of the RTCs assessed sustainability as an implementation outcome, hence, understanding the key drivers of sustainability of relational health interventions is limited. Additionally, few studies reported implementation measures of feedback from participants or stakeholders related to issues such as acceptability and appropriateness of the intervention which could help to refine interventions and improve their effectiveness (Pinto et al., 2024).

Pinto et al. (2024) who reviewed 145 studies (53 interventions) of community-based parenting programs globally also found that fidelity (primarily adherence) was the most common implementation outcome reported, followed by acceptability, with other implementation outcomes substantially less reported. Wolfenden et al. (2019) reviewed 40 community-based trials of chronic disease prevention interventions that tested an implementation strategy and also found that the reporting of implementation outcomes beyond fidelity was limited. The findings from this review and others (Pinto et al., 2024; Wolfenden et al., 2019) suggests that the reporting of the full range of implementation outcomes across intervention fields is scarce and there are opportunities for improvement to measure and report implementation outcomes beyond fidelity, but also more specific dimensions of fidelity.

While reporting checklists such as CONSORT (2010) provide limited explicit guidance (notwithstanding item 5—the description of how the intervention was administered) for how to systematically report a hybrid intervention effectiveness and implementation design and impacts, a growing number of studies provide guidance and examples (e.g.: (Dix et al., 2012)). Ttofi and Farrington (2011) suggest, for example, that it is difficult to determine the moderating effect of an intervention’s implementation on outcomes if studies fail to provide robust implementation data like fidelity. They found that program duration and intensity (dose) for students and teachers was one of the main factors associated with a significant decrease in rates of bullying and victimisation. It is encouraging that some of the studies in this review, that included implementation fidelity as part of their effectiveness assessments, found greater positive changes in relational health outcomes when the dosage was as intended (Botvin et al., 2006; Dawson-McClure et al., 2015; Rincón et al., 2018) and no effect when the intervention was poorly implemented (Kiviruusu et al., 2016; Linhares et al., 2022), providing insight of the importance of an intervention’s implementation to improving health outcomes.

### Validity of Implementation Measures and Data Collection Methods

Most studies reviewed conducted minor fidelity analyses or reporting, not part of effectiveness evaluation, to: a) show if the intervention’s fidelity was high and therefore, not a factor in their results; or b) explain why the intervention showed only small effects (i.e., high variation in implementation). Some studies presented detailed fidelity data in figures and tables and clearly described measurement tools (e.g.: Bierman et al., 2014; Bradshaw et al., 2012), however, studies more commonly reported brief attendance and participation rates. These fidelity data were often reported in the study’s methods section, not results, without clear description on how they were collected, measured or monitored throughout the study. Although not specified in this review, adaptions made to interventions by those implementing them are just as important to consider as fidelity (Toomey et al., 2020). Understanding if and how practitioners have adapted different components of an intervention, may reveal ways to practically strengthen an intervention’s implementation to local needs, whilst still achieving improvement in outcomes. This is particularly important to know before scaling interventions and requires a clear delineation and assessment of the intervention’s core components and the implementation strategies (e.g., See StaRI checklist (Pinnock et al., 2017). Without measures of fidelity and other implementation outcomes, most of the relational health RCTs reviewed were only able to conclude, at best, if their intervention was effective within a specific context.

Most of the reviewed RCTs developed bespoke fit-for-purpose process evaluation questions in surveys or checklists (to measure the relational health intervention’s unique characteristics and content) to be used by independent observers or those delivering the intervention (e.g., teachers), which made it difficult to compare and aggregate relational health and their implementation findings across studies. The lack of psychometrically validated or standardised measures for assessing implementation may have complicated efforts to determine what constitutes "good" implementation and how to replicate successful relational interventions in other settings (Akiba et al., 2022; Lortie-Forgues & Inglis, 2019). Although Implementation Science is a newly establishing field with much future research needed to develop and validate measures, they are available, yet their use in this review was lacking. More than a half of the RCTs reviewed relied on self-reported implementation measures (e.g., facilitator checklists, participant logs, attendance rates), which may have limited validity due to social desirability bias, leniency bias or other potential biases such as a respondent’s mood or motivation (Podsakoff et al., 2003). Whilst the practicalities of collecting fidelity data within real-life services and settings and cost of using independent observers can present challenges, the use of standardised questions and tools will enhance validity and reliability of findings, even with the necessary tailoring to the unique intervention.

While 12 studies did not mention implementation, some RCTs (26 studies) did provide some discussion (albeit not analyses) of their implementation, at least to acknowledge more research was needed (The Multisite Violence Prevention Project, 2014); (Volkaert et al., 2021). Remarkably, of the RCTs that didn’t include implementation measures, none described this as a limitation, despite the growing recognition that variations in implementation quality may explain inconsistencies in outcomes or small effect sizes (Bierman et al., 2014) (Hull et al., 2021). Studies which found smaller than expected effect sizes, for example, may have experienced implementation failure, i.e., the intervention was not implemented in a way that is likely to produce the desired effect and a potential Type 3 Error (Stame, 2010), such as the incomplete or inconsistent delivery of intervention components.

### Use of Implementation Theory and Frameworks

Research is most effective when guided by theory (Tabak et al., 2012). Similar to other research showing poor use of implementation theories and frameworks in intervention efficacy and effectiveness testing (Strifler et al., 2018; Wang et al., 2023), none of the RCT studies reviewed reported using an established implementation theory or framework such as RE-AIM (Glasgow et al., 2019) or The Diffusion of Innovations Theory (Rogers & Rogers, 2003). While this finding may be a consequence of the relative infancy of implementation science (Chambers & Emmons, 2024) and journal-imposed word limits, 50% of the reviewed RCTs were relatively recent, published in the past 10 years.

Implementation frameworks and theory are needed to guide the implementation process and to determine which implementation strategies work when, for whom, and under what conditions (Tabak et al., 2012). It can also help to understand the complexities of real-world interventions, especially in terms of their scalability and sustainability (Birken et al., 2017). Future relational health intervention research should focus on applying and testing existing implementation theories and frameworks such as the meta-theoretical Consolidated Framework for Implementation Research (CFIR) (Damschroder et al., 2022) to improve consensus in implementation language, construct validity and cross-study comparisons (Martinez et al., 2014).

### Limitations

Some limitations of this review should be noted. Firstly, the findings from this secondary analysis are tempered by the search strategy employed by Allen et al. (2026). Only the RCTs selected for review (i.e., universal relational health interventions that are impact focussed) were revisited, excluding other research designs. No contact was made with the authors to seek additional implementation-related publications related to the intervention they were testing, even if additional publications were referred to by the authors (e.g., Chikwari et al., 2025; Debnam et al., 2024; Fonagy et al., 2009; Mageau et al., 2022). This action was taken to reduce the potential publication bias introduced due to differential responses from the authors. Hence, studies that addressed the implementation of relational health interventions tested in these RCTs may have been excluded if they did not also report on relational health outcomes, which in turn may skew our understanding of the full scope of research in this area.

While this review shows only some relational health RCTs collected intervention implementation-related data, identifying each study’s reference to these outcomes was at times difficult due to limited consensus and inconsistent use of key implementation constructs (Martinez et al., 2014). Varied, and sometimes conflicting, descriptions of implementation outcomes, especially within complex community interventions, meant references to these outcomes may have been overlooked within the studies reviewed or allocated to an outcome category not intended by the authors (e.g. intensity-adjusted = dose; attendance = dose (received); satisfaction = acceptability).

Given this review focused on papers reporting RCTs, rather than relational interventions per se (e.g., Good Behaviour Game; Coping Power; PROSPER), the inclusion of multiple papers measuring the same relational health intervention may have skewed the proportion of studies in the overall review that did and did not address implementation. More established relational interventions may, due to the stage of their research for example, have had more opportunities to use concurrent measures of effectiveness and implementation. However, interventions with more than three studies in the review (9 interventions) were mapped by the authors against the significance of their reporting of implementation outcomes and type of trial design and no association was found, suggesting this was not the case in this analysis. For example, only one intervention with multiple studies was a Type 3 trial (Communities That Care) and three interventions with multiple studies were a Type 2 trial (Triple P, The Great Student Curriculum and Great Teacher Program and KiVa). Interestingly, Allen et al. (2026) found that many of the relational interventions tested were already widely implemented, but few were evaluated using a randomised design.

Population-based community interventions were more likely to assess implementation over longer periods of time, possibly due to their complexity and the time required to undertake community-led initiatives at-scale. The reporting of implementation outcomes however was less than expected in these scaled studies potentially due to their comprehensiveness and word limits applied by journals which may have led authors to describe their implementation in other publications that did not meet the scoping reviews’ inclusion criteria.

## Conclusion

This review found that high quality research addressing relational health has, in general, failed to systematically consider implementation outcomes and their influence on relational outcome variables when evaluating the efficacy or effectiveness of interventions using RCTs. Without robust implementation data, particularly fidelity, it is difficult to determine whether an intervention's success or failure is due to the intervention itself or the way it was implemented. Furthermore, without clear understandings and details of implementation factors and outcomes during trial testing, the replicability and scalability of the intervention are unknown. This potentially wastes critical resources and interventions that are not acceptable to the families and communities they aim to support. The findings highlight the need for a more detailed application of implementation science within relational health intervention trials, with more consistent terminology, theory, methodology, measurement and reporting of implementation outcomes across studies.

## Actionable Insights

This review identified several research gaps. The following recommendations focus on the importance of using the available frameworks and validated measures to understand the interaction effects between relational health interventions and their implementation, to improve relational health intervention outcomes and enhance the pace of their scaling and translation into policy and practice (Fernandez et al., 2019).

1. Use Study Designs and Analyses that Account for and Capture Implementation Processes

Develop appropriate methodologies for assessing implementation outcomes for relational health interventions, including multidimensional approaches. New trial designs need to account for and test implementation processes and outcomes alongside relational health outcomes (E.g., conduct both Intention-To-Treat and Treatment-On-the-Treated one analyses). Hybrid implementation-effectiveness trials can provide more rapid evidence and fast-track knowledge translation, helping to understand how and why certain interventions lead to specific outcomes and how interventions perform not only in controlled settings (e.g., efficacy studies) but also in real-world environments where they need to be sustained, adapted, and scaled.

2. Routinely Incorporate Measures of Implementation Outcomes

Carefully measure and report a range of implementation outcomes in relational health interventions trials. This may involve assessing the intervention’s acceptability, adoption, appropriateness, cost, feasibility, fidelity, penetration and sustainability. Accurate measurement helps avoid evaluating the effects of an intervention as described rather than as delivered, reducing the likelihood of Type 3 error and ensuring effective implementation. It also helps to improve effectiveness by refining interventions that are more closely aligned to the needs and context of the children, families and communities they support.

3. Assess Multiple Dimensions of Fidelity

Implementation fidelity assessment should consider both adherence (alignment with the intervention protocol) and quality of delivery. Relational health studies need to be designed with implementation fidelity measures as a primary variable of interest (e.g., dose, adherence, quality of delivery, participant responsiveness, and program differentiation) to provide sufficient statistical power for analyses and to investigate how interventions can be adapted to different contexts without compromising core intervention components, ensuring that flexibility and fidelity are balanced.

4. Use Standardised Measures of Implementation Outcomes

Use psychometrically robust, standardised measures for monitoring intervention outcomes based on established implementation theory. This includes combining and interpreting different forms of implementation outcome data to determine their predictive ability in relational health interventions.

5. Identify Active Ingredients for Implementation

Use implementation science to systematically identify the active ingredients in relational health interventions. RCTs should identify the most critical strategies that define these interventions and determine which elements contribute most significantly to effective delivery and meaningful outcomes. This includes understanding the threshold levels of intervention fidelity and how individual contexts and cultural settings influence relational health intervention design and implementation.

6. Report Implementation Outcomes

Improve and standardise the clarity of reporting of implementation outcomes in relational health RCTs (Lengnick-Hall et al., 2022). Extend reporting frameworks like CONSORT to guide the reporting of implementation outcomes and encourage journals to allow sufficient word limits to report intervention implementation.

## Supplementary Information

Below is the link to the electronic supplementary material.

Supplementary Material 1

Supplementary Material 2

## Data Availability

No datasets were generated or analysed during the current study.
