# Supplementary material for: Opportunities to Improve the Implementation of Relational Health Interventions for Children and Families: A Secondary Analysis of a Scoping Review
Source: Clin Child Fam Psychol Rev. 2026 Apr 30;29(2):323–49. doi: 10.1007/s10567-026-00567-5 (PMC13282296; doi:10.1007/s10567-026-00567-5)
Supplement: Supplementary file 1 — Supplementary Material 1 [file 10567_2026_567_MOESM1_ESM.pdf]

Journal
Clinical Child and Family Psychology Review

Title
Opportunities to improve the implementation of relational health interventions for children and families: A secondary analysis of a scoping review

Pearce, Natasha.[1], *Cross, Donna.[1,2,3], Francis, Jacinta.[1,2,3], Sae-Koew, Jonathan H.[1], Godley, Alexander M.[2], Attwell, Caitlin.[2], Evans-Whipp, Tracy[4], Allen, Jacqueline.[5], Homel, Ross.[5], *Olsson, Craig A.[4,6] & *the Australian Early Relational Health Network*

[1] The Kids Research Institute Australia, Perth Children's Hospital, Western Australia, Australia.
[2] The University of Westen Australia, Western Australia, Australia.
[3] Edith Cowan University, Western Australia, Australia.
[4] SEED Centre for Lifespan Research, School of Psychology, Faculty of Health, Deakin University, Victoria, Australia.
[5] Griffith Criminology Institute, Griffith University, QLD, Australia.
[6] Murdoch Children's Research Institute, Centre for Adolescent Health, Department of Paediatrics, The University of Melbourne, Royal Children's Hospital Campus, Victoria
*Joint senior authors

Corresponding author:
Dr Natasha Pearce, Honorary Research Associate, The Kids Research Institute Australia, Western Australia, Australia. natasha.pearce@thekids.org.au

Online Resource 1: Intervention characteristics of included studies by implementation data collection methods and measures

**Data Collection Method**	N included/% Total n=153	N of Studies*	N of Interventions*	Age Group %	Level of Ecological System %	Setting %
Participant self-report survey	22 (14)	19	17	9% Early childhood<br>55% Childhood<br>32% Adolescence<br>5% Young adult	82% Microsystem<br>14% Microsystem & Mesosystem<br>23% Microsystem, Mesosystem & Exosystem	9% Early childcare<br>64% School<br>5% School and community<br>9% Community<br>5% Primary care<br>5% Multiple community settings<br>5% Online
Interview	2 (1)	2	2	100% Childhood	100% Microsystem	50% School<br>50% Community
Facilitator self-report checklist/log	43 (28)	36	28	7% Early childhood<br>77% Childhood<br>16% Adolescence	86% Microsystem<br>11% Microsystem & Mesosystem<br>2% Microsystem, Mesosystem & Exosystem	11% Early childcare<br>75% School<br>2% School and community<br>11% Community
Independent observer	44 (29)	38	33	9% Early childhood<br>57% Childhood<br>32% Adolescence<br>2% Childhood & Adolescence	89% Microsystem<br>7% Microsystem & Mesosystem<br>5% Microsystem, Mesosystem & Exosystem	14% Early childcare<br>59% School<br>5% School and community<br>5% School and home<br>16% Community<br>2% Laboratory
Study records	29 (19)	25	24	10% Early childhood<br>62% Childhood<br>24% Adolescence<br>3% Childhood & Adolescence	77% Microsystem<br>3% Microsystem & Exosystem<br>20% Microsystem & Mesosystem	13% Early childcare<br>53% School<br>3% School and community<br>7% School and home<br>13% Community<br>10% Multiple community settings
No detail	13 (9)	13	12	55% Childhood<br>36% Adolescence<br>9% Childhood and Adolescence	82% Microsystem<br>9% Exosystem<br>9% Microsystem, Mesosystem & Exosystem	55% School<br>9% School and community<br>9% School and home<br>18% Community<br>9% Laboratory and online

**Data Collection Measures**	N included/% Total n=153	N of Studies*	N of Interventions*	Age Group %	Level of Ecological System %	Setting %
Validated	0	0	0	NA	NA	NA
Developed specific for study	120 (77)	66	57	7% Early childhood<br>68% Childhood<br>23% Adolescence<br>3% Childhood and Adolescence<br>1% Young adult	84% Microsystem<br>1% Exosystem<br>13% Microsystem & Mesosystem<br>2% Microsystem, Mesosystem & Exosystem	9% Early childcare<br>67% School<br>3% School and community<br>2% School and home<br>16% Community<br>1% Primary care<br>1% Multiple community settings<br>1% Online<br>1% Laboratory and online
No detail of implementation measures	33 (23)	27	25	13% Early childhood<br>53% Childhood<br>34% Adolescence	85% Microsystem<br>3% Microsystem & Exosystem<br>3% Microsystem & Mesosystem<br>9% Microsystem, Mesosystem & Exosystem	19% Early childcare<br>47% School<br>6% School and community<br>9% School and home<br>6% Community<br>9% Multiple community settings<br>3% Laboratory

Note: * Multiple data collection methods and measures possible per study/intervention
