# Supplementary material for: Opportunities to Improve the Implementation of Relational Health Interventions for Children and Families: A Secondary Analysis of a Scoping Review
Source: Clin Child Fam Psychol Rev. 2026 Apr 30;29(2):323–49. doi: 10.1007/s10567-026-00567-5 (PMC13282296; doi:10.1007/s10567-026-00567-5)
Supplement: Supplementary file 2 — Supplementary Material 2 [file 10567_2026_567_MOESM2_ESM.pdf]

Journal

Clinical Child and Family Psychology Review

Title

Opportunities to improve the quality implementation of relational health interventions for children and families: A secondary analysis of a scoping review

Pearce, Natasha.[1], *Cross, Donna.[1,2,3], Francis, Jacinta.[1,2,3], Sae-Koew, Jonathan H.[1], Godley, Alexander M.[2], Attwell, Caitlin.[2], Evans-Whipp, Tracy[4], Allen, Jacqueline.[5], Homel, Ross.[5], *Olsson, Craig A.[4,6] & *the Australian Early Relational Health Network*

[1] The Kids Research Institute Australia, Perth Children's Hospital, Western Australia, Australia.
[2] The University of Westen Australia, Western Australia, Australia.
[3] Edith Cowan University, Western Australia, Australia.
[4] SEED Centre for Lifespan Research, School of Psychology, Faculty of Health, Deakin University, Victoria, Australia.
[5] Griffith Criminology Institute, Griffith University, QLD, Australia.
[6] Murdoch Children's Research Institute, Centre for Adolescent Health, Department of Paediatrics, The University of Melbourne, Royal Children's Hospital Campus, Victoria
*Joint senior authors

Corresponding author:
Dr Natasha Pearce, Honorary Research Associate, The Kids Research Institute Australia, Western Australia, Australia. natasha.pearce@thekids.org.au

Online Resource 2: Community- or Population-level Intervention Trials

Table 5: Community- or Population-level Intervention Trials and Implementation (19 studies; 9 interventions)

Intervention name	Country	Intervention Aim	Intervention Type	Age Group	Design	Relational Health Outcomes*	Trial Type	Implementation Outcomes	Data collection methods/ measures
A Better Chance Welfare Reform Program[1]	USA	State welfare reform to incentivise parental employment	Welfare reform	Childhood and adolescence (children < 18)	Random assignment of cases to new rules	Child abuse	Type 1	Fidelity - Dose	Doesn't state; specific to study
Communities that Care (CTC Pennsylvania study)[2]	USA	Improve behavioural health of adolescents across community	Community prevention system	Adolescence	120 communities in Pennsylvania	Family relationships, peer relationships, school climate, community cohesion and social capital	Type 3	Fidelity - Dose	Participant self-report survey; specific to study
Communities that Care (CTC Australia)[3]	Australia	Improve behavioural health of adolescents across community	Community prevention system	Adolescence	Four local government areas plus comparison communities	Protective factors	Not included	-	-
Communities that Care (CTC Community Youth Development Study)[4-6]	USA	Improve behavioural health of adolescents across community	Community prevention system	Adolescence	24 towns in seven states, matched within state	Aggression and violence	Discussion only [4]<br>Not included [5]<br>Discussion only [6]	-	-
Learning to Read in a Healing Classroom[7]	Democratic Republic of the Congo	Promote academic and socioemotional outcomes through social and emotional learning practices	Classroom-based curriculum	Childhood (grades 2-4)	40 clusters of schools in four educational subdivisions within a province	School climate, peer relationships	Discussion only	-	-
Triple P (Every Family study)[8]	Australia	Enhance parental competence and reduce dysfunctional parenting; improve child wellbeing; through five levels of intervention of increasing intensity	Parenting intervention	Childhood (Ages 4-7)	All parents of 4-7 year-old children in ten communities in Brisbane, QLD; ten comparison communities elsewhere	Parenting, aggressive and disruptive behaviour, social competence	Type 1	Fidelity - Dose	Study records; doesn't state
Triple P (U.S. Triple P System Population Trial)[9]	USA	Enhance parental competence and reduce dysfunctional parenting; improve child wellbeing; through five levels of intervention of increasing intensity	Parenting intervention	Childhood (Families with at least one child aged under 8)	18 counties within a state randomised to control and treatment	Child abuse	Type 1 [9]	Fidelity - Dose	Study records; doesn't state

Intervention name	Country	Intervention Aim	Intervention Type	Age Group	Design	Relational Health Outcomes*	Trial Type	Implementation Outcomes	Data collection methods/ measures
The Fast Track Promoting Alternative Thinking Strategies (PATHS) Curriculum[10]	USA	Promote social and emotional competence	Classroom-based curriculum	Childhood (grades 1-3)	Approx. 12 elementary schools in each of three communities across three US states	Aggression and violence, aggressive and disruptive behaviour, social competence	Type 1	Fidelity - Dose	Facilitator self-report checklists/log; specific to study
PROSPER[11-15, 17]	USA	Decrease adolescent problem behaviours	Community prevention system	Adolescence (6th and 7th grade)	28 school districts in two states	Aggressive and disruptive behaviour, peer relationships, family relationships, parenting, intimate partner relationship, intimate partner violence, risky sexual behaviours, community cohesion and social capital	Discussion only [11 – 12, 17]<br>Type 1 [13 – 15]	Fidelity - Adherence to content [13 - 15]	Independent observer; doesn't state [13]<br>Facilitator self-report checklist/log; Specific to study [14]<br>Doesn't state; Doesn't state [15]
Ujana Salama[16]	Tanzania	Reduce risky sexual behaviour and HIV risk	Group-based sessions for children	Adolescence (ages 14-19)	130 villages randomised to treatment and control	Risky sexual behaviours	Type 2	Fidelity - Dose<br><br>Fidelity - Adherence to content	Study records; Doesn't' state<br>Independent observer; Specific to study
Community Wide Initiative [18]	USA	To reduce population rates of teen pregnancy	Community prevention system	Adolescence (ages 15-19)	Intervention was in 10 geographically defined communities with population over 100,000. Synthetic control communities were constructed from 10 comparison communities	Risky sexual behaviours (teen pregnancy)	Discussion only	-	-
Safe and Health Communities Initiative (SHCI) [19]	USA	To reduce population rates of child sexual abuse	Community prevention system	Children and adolescents under 18	Intervention was in 5 counties in Pennsylvania. A synthetic control condition was constructed using 5 comparison counties	Child abuse and maltreatment (child sexual abuse)	Discussion only	-	-

[1](Fein & Lee, 2003) [2](Feinberg et al., 2010) [3](Toumbourou et al., 2019) [4](Hawkins et al., 2012) [5](Oesterle et al., 2014) [6](Oesterle et al., 2018) [7](Aber et al., 2017) [8](Sanders et al., 2008) [9](Prinz et al., 2009) [10] (Conduct Problems Prevention Research Group, 2010) [11](Chilenski et al., 2014) [12](LoBraico et al., 2022) [13](Osgood et al., 2013) [14](Redmond et al., 2009) [15](Spoth et al., 2017) [16](Waidler et al., 2022) [17](Bai et al., 2025) [18](Tevendale et al., 2024) [19](Noll et al., 2025)

Note: *Comprises study outcomes included in the present review.
